# *Spirulina platensis* as a growth booster for broiler; Insights into their nutritional, molecular, immunohistopathological, and microbiota modulating effects

**DOI:** 10.1186/s12917-023-03858-z

**Published:** 2024-01-05

**Authors:** Samar H. Abdelfatah, Aya M. Yassin, Marwa S. Khattab, Ahmed S. Abdel-Razek, Adel H. Saad

**Affiliations:** 1 Department of Nutrition and Clinical Nutrition, Faculty of Veterinary Medicine, Cairo, University, Giza, 12211 Egypt; 2 Department of Biochemistry and Molecular Biology, Faculty of Veterinary Medicine, Cairo University, Giza, 12211 Egypt; 3 Department of Pathology, Faculty of Veterinary Medicine, Cairo University, Giza, 12211 Egypt; 4 Microbial Chemistry Department, Genetic Engineering and Biotechnology Research Division, National Research Center, Dokki-Giza, Egypt; 5 Nutrition and Clinical Nutrition Department, Faculty of Veterinary Medicine, Matrouh University, Matrouh, Egypt

**Keywords:** Broiler, *Spirulina platensis*, Growth performance, Gut health biomarker, And Antioxidant

## Abstract

**Background:**

The present study is designed to assess the effect of adding various doses of *Spirulina platensis* (SP) on broiler chicken growth performance, gut health, antioxidant biomarkers, cecal microbiota, histopathology, and immunohistochemistry of inducible nitric oxide synthase (iNOS). 240 male Cobb 500 broiler chicks (1 day old) were placed into four groups (sixty birds/group), then each group was further divided into three replicates of 20 chickens each for 35 days. Birds were allocated as follows; the 1st group (G1), the control group, fed on basal diet, the 2nd group (G2): basal diet plus SP (0.1%), the 3rd group (G3): basal diet plus SP (0.3%), and the 4th group (G4): basal diet plus SP (0.5%).

**Results:**

Throughout the trial (d 1 to 35), SP fortification significantly increased body weight growth (BWG) and feed conversion rate (FCR) (*P* < 0.05). Bursa considerably increased among the immunological organs in the Spirulina-supplemented groups. Within SP-supplemented groups, there was a substantial increase in catalase activity, blood total antioxidant capacity, jejunal superoxide dismutase (SOD), and glutathione peroxidase (GPX) activity (*P* < 0.05). Fatty acid binding protein 2 (FABP2), one of the gut barrier health biomarkers, significantly increased in the SP-supplemented groups but the IL-1β gene did not significantly differ across the groups (*P* < 0.05). Different organs in the control group showed histopathological changes, while the SP-supplemented chicken showed fewer or no signs of these lesions. The control group had higher levels of iNOS expression in the gut than the SP-supplemented groups (*p* < 0.05). Cecal *Lactobacillus* count significantly elevated with increasing the rate of SP inclusion rate (*p* < 0.05).

**Conclusion:**

Supplementing broiler diets with SP, particularly at 0.5%, can improve productivity and profitability by promoting weight increase, feed utilization, antioxidant status, immunity, and gastrointestinal health.

## Background

Many nutritional strategies have been developed to enhance the genetic potential of broiler chickens, including integrating antibiotics into feed. According to Sugiharto [1], the use of antibiotics in the feed may result in broiler production improvements in growth and feed efficiency as well as a reduction in morbidity and mortality. However, these additives were banned due to the rise of bacterial resistance to antibiotics in both humans and animals. In practice, the removal of synthetic antibiotics from broiler meals caused challenges to the broilers' health and performance [2]. Therefore, using alternative growth promoters to replace antibiotics for broilers was fateful for food safety issues and durable broiler production.

Microalgae including *Chlorella Vulgaris*, *Schizochytrium*, and *Spirulina platensis*, which have good nutritional and functional properties that may be beneficial for broiler chickens, have recently attracted a lot of interest from poultry nutritionists [3]. Among the many benefits of *S. platensis* are that it provides essential amino acids, and fatty acids, as well as vitamins, pigments, minerals, and phenolic acids [4, 5]. In broiler diets, *S. platensis* can replace up to 15% of common protein sources without a negative effect on their growth performance or meat quality [6, 7]. Many studies suggest that *Spirulina* has beneficial effects through its hypolipidemic action, antioxidant, immunostimulatory, hepatoprotective, and anticancer activity also it can combat inflammation, viral, and microbial infection in different experimental animals [4, 8–11]. It also increases intestinal lactobacilli and lessens the nephrotoxic effect of heavy metals and medications, as well as protects against radiation [12]. Moreover, *Spirulina* and its dried form contain several bioactive compounds, which are supposed to contribute to its beneficial effects, like; phenolic acids, flavonoid gamma-linoleic acid, β-carotene, phycocyanin, chlorophyll, saponin, triterpenoid, and steroid [13]. *Spirulina* fortification has been studied extensively for its potential to enhance the well-being and productivity of animals and poultry under typical environmental circumstances [14–16]. Also, *Spirulina* showed its positive effects on poultry under heat-stress environmental conditions [17, 18].

Animal growth performance depends on proper digestion, feed absorption, and gut barrier functioning. A crucial line of defense against environmental foreign antigens is healthy gut development and its mucosal modulations [19] whereas coccidiosis, insufficiently digested protein, and oxidative stress can all lead to gut barrier breakdown [20–24]. It is still uncertain how dietary *Spirulina* supplementation affects broiler chickens and how it works, and there is no definite effective inclusion dose was reported [25]. So, this investigation hypothesized that *S. platensis* improves the growth performance parameters by modulating gut health biomarkers, and the objective of the current study was to ascertain the effects of adding various amounts of dry *S. platensis* powder to the broiler feed on growth performance, gut health biomarkers, immunological histochemistry, and antioxidant characteristics in broiler chickens.

## Methods

### Ethical approval

All experimental procedures and poultry management were performed following the Institutional Animal Care and Use Committee (IACUC), Faculty of Veterinary Medicine, Cairo University. Approval No. is (Vet CU 23052022465) with relevance to ARRIVE guidelines.

### Experimental design

240, one-day commercial Cobb500 male broiler chicks were purchased from Al-Ahram Company, Giza, Egypt, and were alienated into four groups (60 birds/group). Each group was subdivided into three replicates of 20 birds each. The groups were classified as follows; 1st group (G1) served as control and received a basal diet only (corn-soy based diet), 2nd group (G2) received a basal diet enriched with 0.1% *Spirulina platensis*, 3rd group (G3) received basal diet enriched with 0.3% *Spirulina platensis* and 4th group(G4) received basal diet enriched with 0.5% *Spirulina platensis.*

### Diet and housing

The basal diet composition and analysis are presented in Table 1. Based on the CobbTM Manual's recommendations for nutrient requirements, the diets were formulated (Cobb 500™ 2015) [26]. The *Spirulina platensis* was purchased from the Algal Biotechnology Unit, National Research Centre, Dokki, Giza, Egypt. As part of the rearing process (5 weeks), the chickens were reared on a concrete floor covered with fresh wood shaving bedding with an optimal temperature, humidity, and ventilation as well as a 24-h constant lighting system. Besides receiving a balanced diet that included starter, grower, and finisher. Spirulina was added on top of the diet, and the diet was offered in mash form according to the stage of feeding. The starter diet was offered in fine mash form, while the grower and finisher were offered in coarse mash form. The birds also received fresh and clean water ad libitum. At 5 days of age, all birds received the Hitchner-IB vaccine via eye drops. At 9 days, inactivated H5N1 was administered via subcutaneous injection. At 14 days, Gumboro Intermediate Plus was administered via eye drops, and at 21 days, Lasota was administered via eye drops.

**Table 1:** Basal diet physical and chemical composition

Feed ingredient %	Starter	Grower	Finisher
Yellow corn	**55.81**	**60.9**	**62.9**
Soya bean meal (47%)	**33.3**	**27.8**	**24.4**
Corn gluten meal (60%)	**3**	**3.2**	**4.2**
Methionine	**0.24**	**0.24**	**0.2**
Lysine	**0.18**	**0.24**	**0.16**
Soya oil	**3.42**	**3.71**	**4.32**
Monocalcium phosphate	**1.64**	**1.55**	**1.48**
Limestone	**1.66**	**1.61**	**1.59**
Sodium Chloride	**0.35**	**0.35**	**0.35**
^a^Premix	**0.3**	**0.3**	**0.3**
Toxin binder	**0.1**	**0.1**	**0.1**
Total	**100**	**100**	**100**
*Calculated /chemical analysis*			
Metabolizable Energy (Kcal/kg)	**3008**	**3088**	**3168**
Crude protein%	**21.2**	**19.2**	**18.7**
Crude fat%	**2.65**	**2.7**	**2.77**
Crude fiber%	**3.02**	**2.94**	**2.8**
Calcium%	**0.99**	**0.98**	**0.95**
Non-phytate phosphorus%	**0.49**	**0.43**	**0.42**

^a^Each Kg premix contained Vit. A (1,200,000 IU), Vit. D3 (350,000 IU), Vit. E (4000 mg), Vit. B1 (250 mg), Vit. B2 (800 mg), Vit. B6 (600 mg), Vit. B12 (3.2 mg), Vit. K3 (450 mg), nicotinic acid (4.5 g), Ca pantothenate (1.5 g), folic acid (120 mg), biotin (5 mg), choline chloride (55 g), Fe (3 g), Cu (2 g), Mn (10 g), Zn (8 g), I (120 mg), and Co (40 mg)

### Growth performance parameters

The body weight gain (BWG), Feed consumption (FC), and feed conversion ratio (FCR) were used to evaluate the broiler bird growth performance. The individual body weight (BW) of the broiler was estimated at the experiment beginning and every week thereafter. Weekly FC for each replicate was also estimated to calculate FCR according to [27].

### Evaluation of the Carcass

One day before the end of the study, the broiler birds were fasted overnight and then slaughtered by severing the jugular vein using a sharp knife. After that, the slaughtered birds were de-feathered and eviscerated. The carcass and live weights were estimated and recorded for randomly selected birds where for each group; 3 birds were taken from each replicate with a total no. (9 birds/group). Additionally, the immunological organs (spleen, bursa, and thymus) and internal organs (gizzard, liver, and heart) weights were evaluated and expressed as a percentage of live weight in accordance with [28]. The yield of the breast and thigh muscles was measured [29].

### Samples preparation

Blood specimens were collected from the jugular vein into plain, K2 EDTA tubes. Centrifugation of the blood specimens was performed (3500 rpm for 15 min) for separation of serum and plasma. The separated serum and plasma were stored at − 80°C until analysis. One g of jejunum samples was homogenized in 100mM potassium phosphate (pH 7, containing 2mM EDTA/g tissue) for evaluation of superoxide dismutase (SOD) activity while for glutathione peroxidase (GPx)**,** homogenization of tissues was performed in 50 mM phosphate buffer (pH 7 containing 5mM EDTA and 1mM 2-mercaptoethanol). The supernatants were collected after the centrifugation of homogenates (4000 rpm for 15 min at 4°C) and stored at -80°C until analysis. For RNA extraction, 60 mg of the jejunum specimens were cut and collected on liquid nitrogen and stored at − 80°C.

### Determination of the antioxidant status

Total antioxidant capacity (TAC) (mM/L) in serum and plasma catalase activity (U/L) was determined spectrophotometrically using commercial kits (Bio Diagnostic, Giza, Egypt) at 505, 510 nm; respectively [30, 31]. As well, jejunal SOD and GPX activities (U/gT) were determined using commercial kits (Bio Diagnostic, Giza, Egypt) at 560, 340 nm; respectively [32, 33].

### Quantitative Real-Time RT-PCR of FABP2 and IL-1β

A total RNA purification kit (Jena Bioscience, Germany, Cat. #PP-210S) was used for total RNA isolation following the manufacturer’s instructions. The RNA concentration and purity were ascertained using Nanodrop ND-1000 Spectrophotometer (Thermo Scientific). The total RNA was then reversely transcribed using the Revert Aid First Strand cDNA Synthesis Kit (Thermo Scientific, USA, Cat. #K1622). The level of mRNA expression of each gene was estimated relative to β-actin (ACTB) as an endogenous reference gene through a fluorescence-based real-time detection method using iQ SYBR® Green Supermix (Bio-Rad 1,708,880, USA) according to manufacturer instructions. Primer sequences for fatty acid binding protein 2 (FABP2) and IL-1β were designed according to [34] while the ACTB sequence was designed using Primer 3 software (Table 2). The cycle threshold (Ct) values were obtained using Bio-Rad iCycler thermal cycler and the MyiQ real-time PCR detection system. The cycling protocol was performed at 95°C for 3 min (initial denaturation) and then 40 cycles of denaturation (at 95°C for 15 s), annealing (at 60°C for 30 s), and extending (at 72°C for 30 s). PCR products were tested for specificity by checking the melting curves at the end of each reaction. Each assay was executed in triplicates and no-template negative control (NTC) was included; the expression relative to control was calculated using the equation; 2-ΔΔCT [35].

**Table 2:** Primers used for qRT–PCR

Genes	Forward primer	Reverse primer	Product size	REF./accession No
ACTB	CCCACACCCCTGTGATGAAA	TAGAACTTTGGGGGCGTTCG	177	NM_205518.1
FABP2	AAAGATAATGGAAAAGTACTCACAGCAT	CCTTCGTACACGTAGGTCTGTATGA	77	Chen et al., 2015
IL-1β	CAGCCCGTGGGCATCA	CTTAGCTTGTAGGTGGCGATGTT	59	Chen et al., 2015

*ACTB* Beta-actin gene, *FABP2* Fatty acid binding protein 2 gene, *IL-1 β* Interleukin -1β

### Histopathological evaluation

Tissue specimens from the intestine, liver, spleen, bursa, and thymus were fixed in 10% neutral buffered formalin. Then tissues were treated with a paraffin embedding technique and sectioned 3-4µm thick using a rotatory microtome. Tissue sections were deparaffinized, stained with hematoxylin and eosin stain, examined by a light microscope (Olympus BX43), and photographed by a digital camera (Olympus DP27).

### Immunohistochemistry

Inducible nitric oxide synthetase (iNOS) was detected in paraffin-embedded tissue sections using polyclonal rabbit anti-iNOS (1:200) (bs-2072R Bioss, Beijing, China) after deparaffinization, rehydration, and antigen retrieval by sodium citrate buffer (pH6.0). According to the manufacturer’s protocol, 3 3'-diaminobenzidine (Dako, Carpinteria, USA) was used to create a brown colour after an hour of application of a secondary horseradish peroxidase-conjugated anti-species antibody (Envision, Dako, Carpinteria, USA). Using Image J software, three photographs (200X) per bird were analyzed to determine the percent of positive staining.

### Microbiological analysis of cecal microflora

The lactobacillus and coliform bacterial quantification were carried out following the method reported by [25]. The cecal specimens were aseptically removed and one gram of the cecal sample was then serially diluted (ten-fold dilution) using sterile distilled water. For viable count, 100µl of each dilution was plated on De Man, Rogosa, and Sharpe agar plates for Lactobacillus spp. counting and MacConkey agar plates for coliform bacteria counts. After inoculation, the plates were incubated for 48h at 37°C under anaerobic conditions for MRS plates and aerobic conditions for MacConkey agar plates. After the incubation periods, colonies of the lactobacillus and coliform bacteria were enumerated and expressed as the logarithm of colony-forming units per gram (log10 CFU/g).

### Statistical analysis

The obtained data were statistically analyzed by using SPSS® version 18 software PC (2008) [36]. A one-way analysis of variance (ANOVA) was used to compare the means of several groups and LSD post hoc test was used when there was a significant difference between treatments [37]. Data were presented as mean ± SE. Statistical significance was set at *p* < 0.05.

## Results

### Growth performance indices

Results of growth performance indices (body weight development, body weight gain, feed intake, FCR) are illustrated in (Table 3). The results demonstrated that the chicks fed with 0.3% and 0.5% of *S. platensis* gained more body weight and had a better feed conversion ratio as well as the cumulative body weight growth and FCR were significantly improved compared to the control group (*p* < 0.05).

**Table 3:** Effect of *S. platensis* fortification on the growth performance of broiler birds

	G1	G2	G3	G4	*P* value
Bodyweight (g):					
Initial weight	45	45	46	46.5	
7^th^day	141.19 ± 2	142.72 ± 1.98	143.38 ± 2.08	142.22 ± 2.05	0.90
15^th^day	439.02 ± 9.1	440.27 ± 8.1	440 ± 8	435.41 ± 7.6	0.97
21st day	981.81 ± 12.9 ^b^	978.57 ± 14.36 ^b^	1007.77 ± 8.29 ^ab^	1023.89 ± 11.12 ^a^	0.02
28^th^day	1598.78 ± 18.62 ^b^	1602. ± 17.41 ^b^	1645.83 ± 17.33 ^ab^	1674.28 ± 21.15 ^a^	0.012
35^th^day	2168.38 ± 27 ^b^	2 161.76 ± 30.7 ^b^	2254.30 ± 25.50 ^a^	2289.69 ± 22.34 ^a^	0.001
BWG (g):					
1st – 7th day	101.19 ± 0.69	101.26 ± 1.2	102.72 ± 0.7	101.88 ± 1.2	0.707
7th – 14th day	297.83 ± 6.1	298. ± 6.1	296.61 ± 1.7	293.19 ± 6.7	0.919
14th – 21st day	542.79 ± 16.22^b^	538.19 ± 6.1^b^	567.77 ± 2.4 ^ab^	588.47 ± 6.1^a^	0.016
21st – 28th day	616.96 ± 16.5	623.82 ± 8.3	638.05 ± 8.7	649.87 ± 8.9	0.236
28th – 35th day	569.1 ± 10.49 ^b^	559. ± 6.4 ^b^	608.47 ± 11.8 ^a^	616. ± 7.5 ^a^	0.006
1st – 35th day	2127.88 ± 4.4 ^c^	2120.31 ± 10.6 ^c^	2213.63 ± 7.11^b^	2249.46 ± 3.98 ^a^	0.00
FC (g):					
1st – 7th day	131.11 ± 1.3	129.30 ± 0.8	130.27 ± 1.2	133.05 ± 1.5	0.273
7th – 14th day	381.61 ± 2.5	382.47 ± 7.1	379 ± 6	369 ± 14.9	0.702
14th – 21st day	789.03 ± 2.5	786.25 ± 12.3	798.9 ± 12.3	809.58 ± 2.7	0.388
21st- 28th day	1035.39 ± 7.9	1038.35 ± 3.9	1046.94 ± 7.9	1041.54 ± 6.3	0.666
28th – 35th day	1146.62 ± 15.2	1133.97 ± 14.57	1151.72 ± 1.7	1146.14 ± 10.66	0.754
1st – 35th day	3492.77 ± 8.5	3470.36 ± 22.47	3506.88 ± 11.64	3499.32 ± 14.5	0.413
FCR (feed/gain)					
7th day	1.29 ± 0.007	1.27 ± 0.02	1.26 ± 0.01	1.3 ± 0.02	0.996
15th day	1.28 ± 0.02	1.28 ± 0.03	1.27 ± 0.01	1.26 ± 0.03	0.917
21^st^day	1.47 ± 0.04 ^a^	1.46 ± 0.018 ^ab^	1.4 ± 0.02^ab^	1.37 ± 0.017 ^b^	0.145
28^th^day	1.68 ± 0.03 ^a^	1.66 ± 0.019 ^ab^	1.64 ± 0. 01 ^ab^	1.6 ± 0.02 ^b^	0.169
35^th^day	2.0 ± 0.01^a^	2.0 ± 0.008 ^a^	1.87 ± 0.049 ^b^	1.86 ± 0.018 ^b^	0.004
1st – 35th day	1.64 ± 0.004 ^a^	1.63 ± 0.008 ^a^	1.58 ± 0.005 ^b^	1.55 ± 0.009 ^c^	0.00

G1 Control basal diet, G2 basal diet plus 0.1% S. platensis, G3 basal diet plus 0.3% S. platensis; G4 Basal diet plus 0.5% S. platensis. Data represented as mean value ± standard error (S.E.) where (*n* = 3/ replicate). a,b,c Different superscripts in the same row indicate a significant difference (*P* < 0.05)

### Carcass traits

Results of carcass traits between the control group which fed on basal diet only and *S. platensis* supplemented groups are presented in (Table 4). Results illustrated that chicks fed with 0.5% spirulina had a higher carcass yield %, breast %, and weight of bursa relative to live weight.

**Table 4:** Effect of *S. platensis* fortification on the carcass traits of broiler birds

Items	G1	G2	G3	G4	*P* value
Dressing yield (%)	72.32 ± 0.32 ^b^	71.98 ± 0.48 ^b^	71.91 ± 0.49 ^b^	73.62 ± 0.16 ^a^	0.018
Breast (%)	23.64 ± 0.18^b^	23.72 ± 0.19 ^b^	23.69 ± 0.23^b^	24.97 ± 0.34^a^	0.003
Thigh (%)	28.8 ± 0.25	28.6 ± 0.32	28.8 ± 0.17	28.75 ± 0.19	0.910
Liver (%)	2.10 ± 0.08	2.17 ± 0.09	2.18 ± 0.08	2.16 ± 0.1	0.931
Gizzard (%)	1.97 ± 0.11	2.08 ± 0.1	2.07 ± 0.09	2.07 ± 0.09	0.867
Heart (%)	0.51 ± 0.03	0.53 ± 0.02	0.54 ± 0.03	0.52 ± 0.02	0.877
Spleen (%)	0.11 ± 0.003	0.11 ± 0.003	0.11 ± 0.001	0.10 ± 0.002	0.564
Bursa (%)	0.15 ± 0.004^b^	0.15 ± 0.004^b^	0.15 ± 0.003^b^	0.18 ± 0.004^a^	0.00

*G1* Control—basal diet; G2: basal diet plus 0.1% *S. platensis*; G3: basal diet plus 0.3% *S. platensis*; G4: basal diet plus 0.5% *S. platensis*. Data represented as mean value ± standard error (S.E.) where (*n* = 3/ replicate). ^a,b,c^ Different superscripts in the same row indicate a significant difference (*P* < 0.05)

### The blood and intestinal antioxidant status

**Fig. 1:**
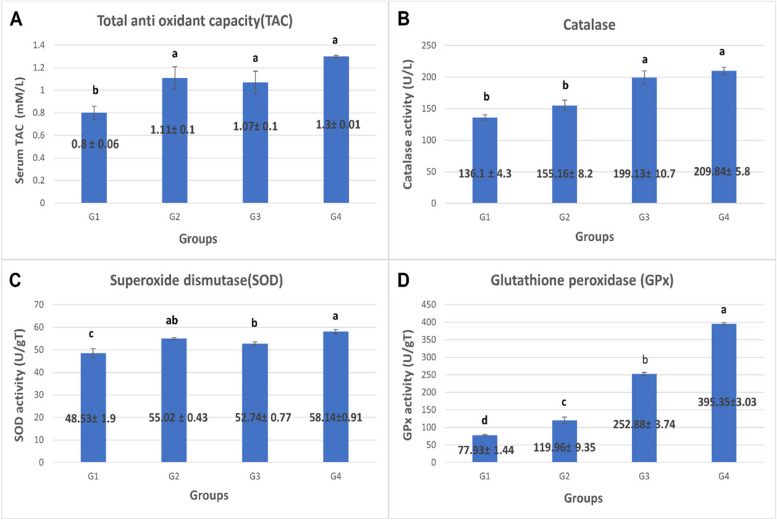
The effect of *S. platensis* on the antioxidant status of broiler chickens. **A** represents the serum TAC (mM/L), (**B**) plasma catalase activity (U/L), (**C**) jejunal SOD activity (U/gt), and (**D**) jejunal GPx activity (U/gt). G1: Control—basal diet; G2 basal diet plus 0.1% *S. platensis*; G3: basal diet plus 0.3% *S. platensis*; G4: basal diet plus 0.5% *S. platensis*). Data represented as mean value ± standard error (S.E.) where (*n* = 3/ replicate). Values with different superscripts are significantly different at *P* < 0.05

Examined serum antioxidant variables were significantly affected by *S. platensis* supplementation of broiler chickens’ diet. As illustrated in Fig. 1, the total antioxidant capacity (TAC) was significantly increased in the Spirulina-supplemented group (with no significance between the different concentrations) than in those fed the basal diet only (*P* = 0.01). While the catalase activity was significantly increased in the groups fed with 0.3% and 0.5% *S. platensis* compared to the control and the 0.1% *Spirulina*-supplemented group (*P* = 0.00). SOD activity showed a significant elevation for all groups supplemented with *S. platensis* than the control one. The 0.5% inclusion rate was recorded as the highest increase followed by 0.3% with no significance between the 0.1% and 0.3% inclusion rates (*P* = 0.002). Glutathione peroxidase (GPx) showed a significant elevation in all *Spirulina*-supplemented groups over the control groups (*P* = 0.00).

### The gut barrier health biomarkers gene expression

**Fig. 2:**
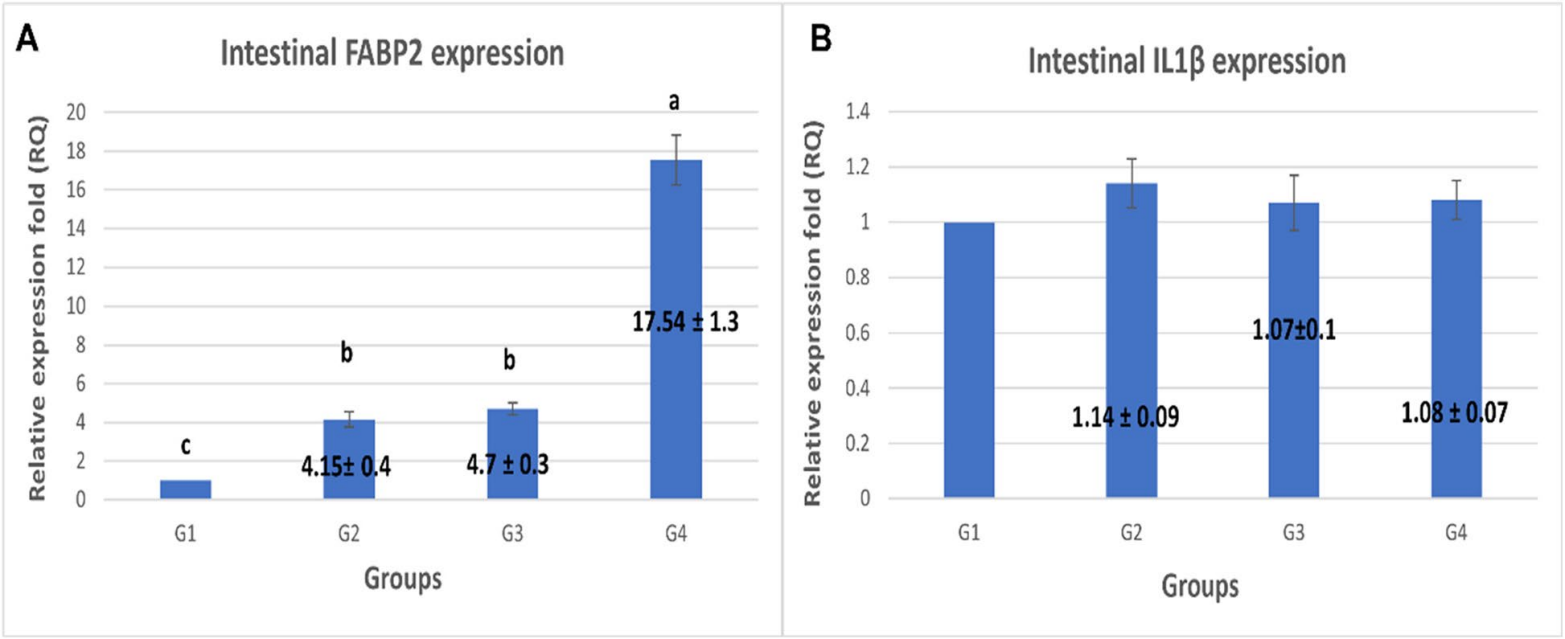
The effect of *S. platensis* supplementation on the relative expression level of jejunal FABP2 and IL1β genes. qRT-PCR results are represented for (**A**) FABP2 gene, (**B**) IL-1β gene. G1: Control—basal diet; G2: basal diet plus 0.1% *S. platensis*; G3: basal diet plus 0.3% *S. platensis*; G4 basal diet plus 0.5% *S. platensis*). Data represented as mean value ± standard error (S.E.) where (*n* = 3/ replicate). Values with different superscripts are significantly different at *P* < 0.05

The relative mRNA expression level for genes that are related to the gut barrier function and inflammation in the jejunum of broilers is shown in Fig. 2. The FABP2 showed significant up-regulation in all *Spirulina*-supplemented groups; 0.1 %,0.3%, and 0.5% by 4.15,4.7 and 17.54 folds, respectively (*P* =0.00). The highest level of expression was recorded for the 0.5% supplemented group. IL1 β as one of the inflammatory biomarkers showed no significant difference in its level of expression between the control and *Spirulina*-supplemented groups (*P*=0.717).

### Histopathological and immunohistochemical findings

**Fig. 3:**
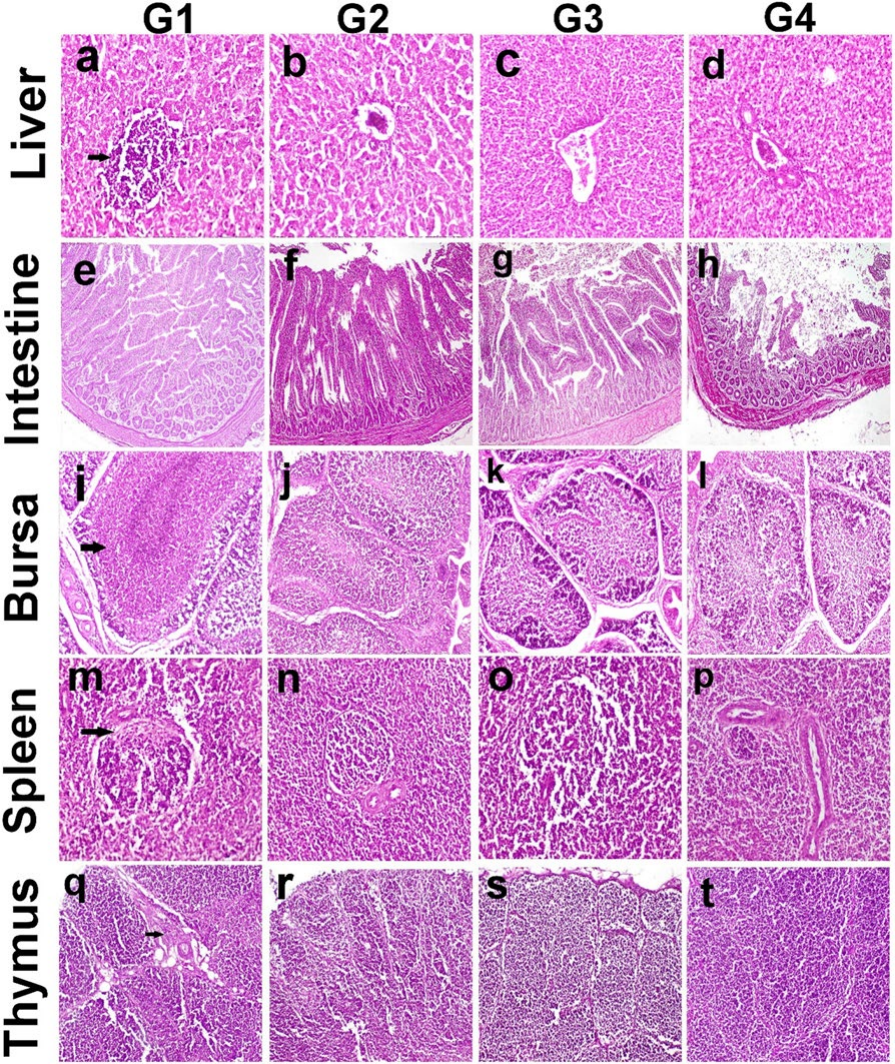
Histopathology of chicken organs in different groups. **a**-**d** Liver, (a) G1(arrow), (b) G2, (c) G3, (d) G4;(X400). (e–h) intestine, (e) G1, (f) G2, (g) G3, (h) G4;(X100). (i-l) bursa (i) G1(arrow), (j) G2, (k) G3, (l) G4;(X400). (m-p) spleen, (m) G1 (arrow), (n) G2, (o) G3, (p) G4;(X 200). (q-t) thymus (q) G1(arrow), (r) G2, (s) G3, (t) G4; (X200). Hematoxylin and eosin stain. G1: Control—basal diet; G2: basal diet plus 0.1% *S. platensis*; G3: basal diet plus 0.3% *S. platensis*; G4: basal diet plus 0.5% *S. platensis*)

In the present study, microscopy of the liver revealed multifocal portal leukocyte infiltration in G1 (Control group) (Fig. 3 a). Whereas in G2, G3, and G4, leukocytic infiltration was absent or only mild infiltration (Fig. 3b, c, d). Microscopy of the intestine in G1 revealed leukocytic infiltration in the submucosa in addition to necrosis, blunting, and adhesion in other sections (Fig. 3e). In G2, the leukocytic infiltration was also observed in the intestinal mucosa (Fig. 3f). In G3, the intestinal villi showed mild diffuse enteritis and decreased lesion severity compared to G1 and G2 (Fig. 3g). In G4, the intestinal histopathological lesion was like those observed in G1 (Fig. 3h). Microscopy of the bursa of Fabricius revealed moderate lymphocytic depletion in lymphoid follicles in addition to heterophilic infiltration in some follicles in G1 (Fig. 3 i). On the other hand, the lymphoid follicles were mildly depleted or almost well populated by lymphocytes with no evidence of heterophilic infiltration in G2, G3, and G4 (Fig. 3j, k, l). Microscopy of the spleen in the control group showed lymphoid depletion and lymphocytolysis whereas spleen in other groups showed normal histological structure (Fig. 3m, n, o, p). Microscopy of the thymus in G1 showed heterophils infiltration in between the thymic follicles whereas it showed normal histological structure in other groups (Fig. 3q, r, s, t).

### Immunohistochemical analysis

**Fig. 4:**
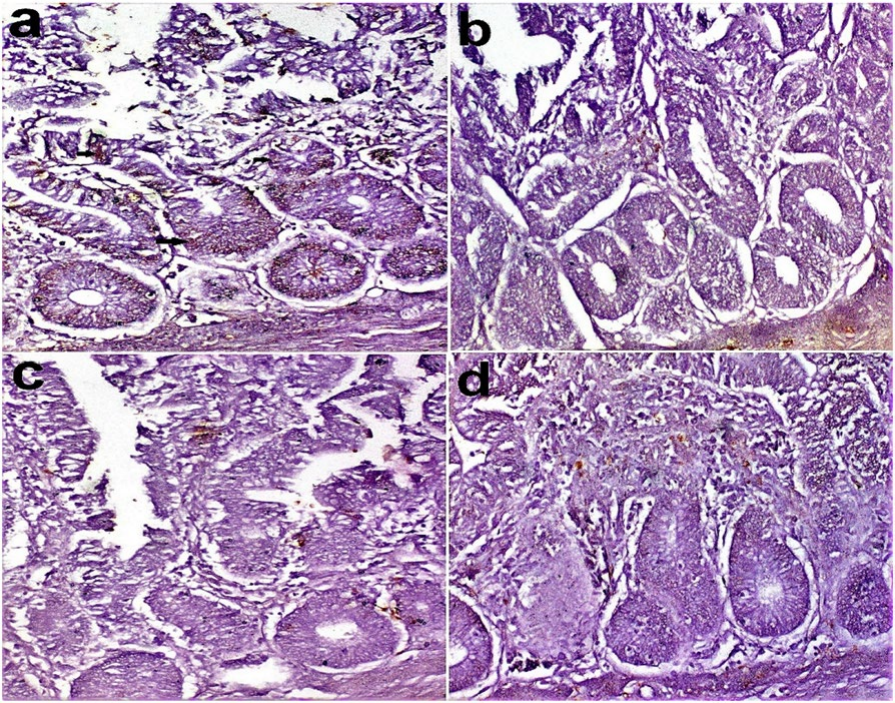
Immunohistochemistry of iNOS in the chicken intestine. **a** G1(arrow), (**b**) G2, (**c**) G3, (**d**) G4. G1: Control—basal diet; G2: basal diet plus 0.1% *S. platensis*; G3: basal diet plus 0.3% *S. platensis*; G4: basal diet plus 0.5% *S. platensis*)

**Fig. 5:**
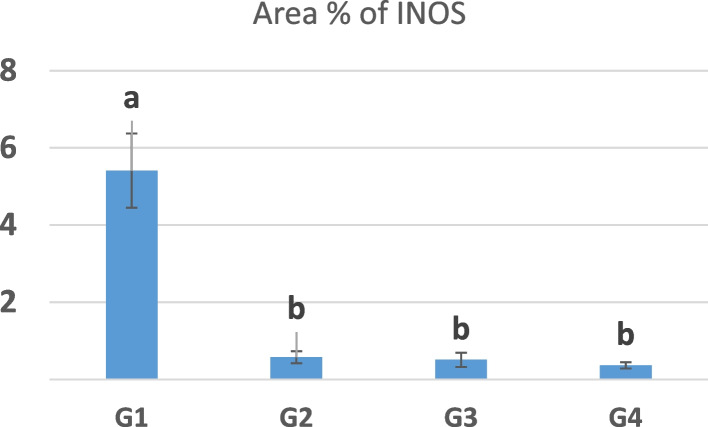
The area % of iNOS expression in the intestine. G1: Control—basal diet; G2: basal diet plus 0.1% *S. platensis*; G3: basal diet plus 0.3% *S. platensis*; G4 basal diet plus 0.5% *S. platensis*). Data represented as mean value ± standard error (S.E.) where (*n* = 3/ replicate). Values with different superscripts are significantly different at *P* < 0.05

Immunohistochemistry of iNOS in the intestine of chicken in different groups revealed the positive expression of iNOS in crypt cells and a few leukocytes in G1. However, its expression was regressed in crypt cells and leukocytes in G2, G3, and G4 Figs. 4 and 5.

### The cecal microflora count

Results for cecal microflora are illustrated in (Table 5), the results revealed a significant elevation in the total colony forming units (CFU) and the coliform count at an inclusion rate of 0.3% and 0.5% of *Spirulina* while there is no significant difference between the control groups and 0.1% *Spirulina* supplemented groups (*P* < 0.05). There was a significant elevation observed in the lactobacillus count for broiler birds fed with different concentrations of *Spirulina* compared to the control group (*P* < 0.05).

**Table 5:** The impact of feeding *S. platensis* supplementation on broilers' cecal microbiota count

Items, log10 cfu/g	G1	G2	G3	G4	*P* value
Total CFU	7.96 ± 0.02^c^	7.71 ± 0.01^c^	8.25 ± 0.005^b^	8.36 ± 0.01^a^	0.00
Coliform	7.56 ± 0.02 ^c^	7.5 ± 0.03 ^c^	7.9 ± 0.014 ^b^	8.0 ± 0.008 ^a^	0.00
lactobacillus	7.13 ± 0.03 ^d^	7.2 ± 0.02 ^c^	7.92 ± 0.01 ^b^	8.05 ± 0.01 ^a^	0.00

*G1* Control—basal diet, *G2* basal diet plus 0.1% *S. platensis, G3*: basal diet plus 0.3% *S. platensis*; G4: basal diet plus 0.5% *S. platensis*). Data represented as mean value ± standard error (S.E.) where (*n* = 3/ replicate). Values in the same row with different superscripts are significantly different at *P* < 0.05

## Discussion

### Growth performance indices

Spirulina's mode of action for promoting growth is not well understood [25]. The current study's findings corroborated prior findings from [38–41] that dietary fortification of spirulina significantly (*P* < 0.05) enhanced body weight, body weight increase, and FCR. The increase in feed utilization efficiency is what accounts for Spirulina's beneficial effects on body weight and BWG. Furthermore, this outcome is consistent with studies by Fathi (2018) and Abd El-Dayem et al. (2021) [42, 43], which found that the addition of Spirulina had a favorable impact on body weight increase and feed conversion ratio. Because spirulina has a great nutritional profile and contains all the required amino acids, vitamin C and antioxidant carotenoids, B Complex, mineral elements, and essential fatty acids, it has a strong positive influence on growth performance parameters. Additionally, spirulina enhances mucosal immunity by encouraging Ig A and Ig E production and nurturing a healthy microbiome [11, 12].

### Carcass traits

The current findings are consistent with those recorded by Fathi (2018) [42], who showed that the addition of spirulina had a considerable favorable influence on slaughter parameters at 38 days of age, except for total giblets (liver, heart, and gizzard), which had been considerably affected. Broiler carcass metrics were greatly enhanced by dietary spirulina supplementation, as demonstrated by Bellof and Alarcon (2013) [44]. Also, Kaoud (2012) [45] showed an improvement in carcass yield %, breast %, and weight of bursa relative to live weight in the *Spirulina*-supplemented group relative to the control group. The increase in carcass yield percentage and breast muscle percentage might be attributed to spirulina's superior nutritional profile, which comprises all essential amino acids. For improved carcass protein and fat reduction, essential amino acids should be included in the diet in a greater proportion than non-essential amino acids. Lysine, which is comparatively more abundant in breast muscle than other amino acids, is important for muscle growth. In breast flesh, lysine makes up around 7% of the protein. One of the essential amino acids for protein synthesis and muscle deposition, lysine has also been shown to be involved in the production of cytokines and the proliferation of lymphocytes, resulting in the best immune system performance in the face of illness [46]. The amelioration in the bursa's relative weight may indicate that spirulina boosts immune system function by enhancing immune cells' and organs' resistance to environmental or infectious agent stress [12].

### The antioxidant status

*Spirulina* antioxidant effect may be associated with the presence of a wide variety of antioxidant components which include ß-carotene, astaxanthin, phycocyanin, phycoerythrin, tocopherol, selenium, phenolic acid sand sulfated polysaccharides such as fucoidans and heterofucans [47–49]. In this study, the Spirulina-supplemented group considerably outperformed the control group in terms of TAC, catalase, GPx, and SOD levels. GPx and SOD are typically considered to function in cells as enzymatic free radical scavengers [50]. *Spirulina* supplementation has been shown to modify the redox status of the broilers in normal and challenging environments; for example, Park et al., 2018 [25] have reported that GPx and SOD linearly increase in broiler birds fed with *Spirulina* at concentrations (0.25%,0.5%,0.75, and 1%). Dietary *Spirulina* also was reported to ameliorate the heat stress adverse effect on broiler oxidant/antioxidant status by decreasing malondialdehyde (MDA) levels and increasing the activity of SOD and TAC as well as reduced glutathione (GSH) concentration [5, 18, 51]. These results were justified by the scavenging effect of *Spirulina* for the hydroxyl radicals due to its content of C-phycocyanin [52]. As an antioxidant, C-phycocyanin is nearly 16 times more potent than Trolox (a vitamin E analog) and 20 times more potent than vitamin C [53]. Several phenolic compounds in *Spirulina* may also contribute to its antioxidant activity, separately or in combination. These compounds include salicylic, trans-cinnamic, synaptic, chlorogenic, quinic, and caffeic acids [54].

### The gut barrier health

The gut is considered a selective barrier for absorbing nutrients and excluding undesirable molecules and pathogens [22, 55, 56]. Therefore, maintaining the gut barrier's appropriate function is crucial for the body's overall health and balance as well as for maintaining its defense against external antigens from the environment [19]. When the intestinal barrier is compromised, it is easier for bacteria to transmit their endotoxins, which activates the innate immune system as well as the adaptive immune system [57, 58]. In the present study, two of the gut barrier function and inflammation biomarkers in broiler jejunum have been investigated. Our results revealed that Spirulina supplementation improved the gut barrier health indicated by the increased expression of intestinal FABP2 while the IL1β gene expression as an inflammatory biomarker didn’t change between groups. In agreement with our results, Chen et al., (2015) [34] reported the mRNA expression of FABP2 was decreased while IL1β increased in the jejunal mucosa of GBF (gut barrier failure) group chickens at day 28 in comparison to the control group.

In the small intestine and ileum, respectively, intestinal FABP (FABP2 and FABP6) is expressed at high levels. By modifying crucial lipid-sensitive pathways in human adipocytes and macrophages, they play a part in intestinal inflammatory situations in addition to their function in lipid metabolism [59, 60]. Broiler chickens' abdominal fat content is significantly influenced by lipogenesis and fatty acid transport [61–63] and FABP2 has been found as a particular marker for the proportion of epithelium in both humans and pigs [64]. Additionally, FABP2 was shown to be downregulated in individuals with ischemia/reperfusion-induced intestinal barrier damage [65], proposing its significant role in gut barrier health. One of the key mediators of inflammation, IL-1β is involved in several biological processes, including cell division, proliferation, and death [66]. Increased levels of IL-1β in the intestinal mucosa are a sign of gut barrier failure, intestinal inflammation, and an active intestinal innate immune response [34].

### Histopathological and immunohistochemical findings

The banning of antibacterial drugs increases the occurrence of necrotic enteritis in which the subclinical form is more prominent [67]. The absence of mortality, reduced growth, and reduced feed efficiency are the main clinical findings of the subclinical form. Other alternatives to antibacterial drugs are being investigated to control necrotic enteritis [68]. *Spirulina* is a potential candidate to control spontaneous diseases in chickens as it can modulate immune functions and possess anti-inflammatory properties [69].

In the groups supplemented with *Spirulina*, the hepatic lesions were almost absent compared to the untreated group suggesting an increased disease resistance in chickens. A previous review showed that *Spirulina* could be a useful protein source for poultry diets with no adverse impact on growth performance [70]. Intensive poultry production and restriction of antibacterial drugs would promote the emergence of the disease [71]. Likewise, the current study in which enteritis was conspicuous in the control group. however, the supplementation of *Spirulina* decreased the severity of intestinal lesions in treated groups.

The bursa of Fabricius is the primary lymphoid organ in the chicken which is exposed to environmental antigens through its connection with the cloaca allowing the stimulation of the immune system [72]. Many causes result in bursal lesions and atrophy including infectious causes or mycotoxins [73]. In the present study, lymphoid depletion in some follicles with heterophilic infiltration was observed in the control group and was absent in *Spirulina-*supplemented groups indicating a better immune response with less damage to bursa lymphoid follicles.

The spleen is the major peripheral lymphoid organ in chickens because it lacks lymph nodes. It has a crucial role in the immune responses elicited against acquired antigens of viruses and bacteria [74]. Therefore, the spleen can be exposed to various types of antigens which may alter its histological structure like the current finding in control chickens. On the contrary, the groups supplemented with Spirulina showed a better histological structure of the spleen. An earlier investigation revealed that Spirulina boosted the treated chicken's lymphoproliferative response [75]. Thymus is a primary lymphoid organ in which T-lymphocytes develop, differentiate, maturate, and become selected [76]. Various pathogens can cause thymic atrophy [77]. Mild abnormalities were seen in the thymus of the control group in the current investigation, but the histology of the thymus was unaltered in the groups that received supplemental spirulina.

Nitric oxide (NO), an active messenger molecule, is released by nitric oxide synthetases which are constitutive NO synthases (cNOS) and inducible NO synthases (iNOS) [78]. Production of NO by iNOS worsens gastrointestinal injury due to oxidative stress in mice [79]. Therefore, NO is always associated with intestinal tract injury [80]. iNOS expression increases with inflammation as demonstrated in previous studies, and can be related to the degree of damage in the mucosa of the intestine [80]. On the other hand, the spirulina-supplemented groups exhibited a downregulation in the expression of iNOS in the gut.

### Cecal microflora count

The intestinal microbial environment including cecum was reported to be highly associated with the production performance in broilers [81]. In the present study, cecal microflora was investigated for the evaluation of the effect of *Spirulina* supplementation. Our study revealed that *Spirulina* supplementation led to increasing the cecal *Lactobacillus* count by increasing its inclusion rate while the coliform count was increased at the 0.3% and 0.5% inclusion rates. Numerous studies have investigated how *Spirulina* supplementation affects gut microbiota**,** in laying quails suffering from heat stress; different levels of *Spirulina* supplementation have decreased the *E. coli* population in the ileal content with no difference in the lactobacillus count at d 134 [82]. *Spirulina* supplementation increased cecal Lactobacillus concentration, according to Park et al., (2018) [25], but did not affect the number of coliform bacteria. Furthermore, according to Shanmugapriya et al., (2015) [40], dietary Spirulina promoted Lactic acid bacteria counts in the gut while reducing E. coli levels in the ileal and cecal contents of broiler chicks. The antibacterial effect may be contributed to the presence of various chemicals such as 1-Octadecene,1-Heptadeceane [83], acrylic acid [84] flavonoids, fatty acids, triterpenoids, and phenolic compounds [85, 86]. *Spirulina's* methanolic and aqueous extracts were studied *in-vitro* and shown to inhibit the development of *Candida albicans* while promoting *Lactococcus lactis* [85]. Also, *Spirulina* extract inhibited the growth of *S. aureus*, *E. coli, P. aeruginosa, S. Typhi,* and *K. pneumonia* [87]. Adding 10 mg/mL of dry *Spirulina* into de Man, Rogosa, and Sharpe medium enhanced the growth of *Lactobacillus acidophilus* [88]. In correlation with our results for the antioxidant status, gut health biomarkers, and histopathological findings; the increase in the coliform count didn’t affect the broiler chicken performance and suggested the promoting effect of Spirulina supplementation.

In conclusion, Dietary inclusion of *Spirulina* at 0.1%,0.3%, and 0.5% improved the FCR and BWG. These observed improvements can be justified as *Spirulina* has increased the antioxidant enzyme activities which improved the redox status of the broilers. Also, it has improved the gut barrier health and immunity resulting in good feed absorption and utilization in addition to increased cecal *Lactobacillus* populations. Based on histopathological findings, the broiler’s overall immunity has been improved. Therefore, *Spirulina platensis* dietary supplementation especially at 0.5% may represent a good natural feed additive and hence improve productivity. However, further studies are needed to investigate the underlying molecular modulating effects of *Spirulina* and its usefulness in the stressful production practice in the poultry industry.

## Data Availability

All data generated or analyzed during this study are included in this published article.

## Abbreviations

BWG: Body weight gain
FABP: Fatty acid binding protein
FCR: Feed conversion rate
GPX: Glutathione peroxidase
iNOS: Inducible NO synthases
SOD: Superoxide dismutase
TAC: Total antioxidant capacity
